# Efficacy of fipronil/(*S*)-methoprene/eprinomectin/praziquantel (Broadline^®^) against *Thelazia callipaeda* in naturally infected cats

**DOI:** 10.1186/s13071-021-04983-0

**Published:** 2021-09-15

**Authors:** Stefania Zanet, Simone Morelli, Angela Di Cesare, Stefano Bò, Donato Traversa, Wilfried Lebon, Frederic Beugnet, Giulia Simonato, Ezio Ferroglio

**Affiliations:** 1 Department of Veterinary Sciences, University of Turin, Turin, Italy; 2 Faculty of Veterinary Medicine, University of Teramo, Teramo, Italy; 3 Ambulatorio Veterinario Bo-Ferro, Turin, Italy; 4 Boehringer Ingelheim Animal Health, Lyon, France; 5 Department of Animal Medicine, Production and Health, University of Padua, Padua, Italy

**Keywords:** *Thelazia callipaeda*, Cat, Eprinomectin, Broadline^®^

## Abstract

**Background:**

The present clinical field trial was conducted to assess the efficacy of a broad-spectrum parasiticide spot-on formulation containing eprinomectin (Broadline^®^) against *Thelazia callipaeda* eyeworm in naturally infected cats.

**Methods:**

Fifteen privately owned cats harboring at least one live adult *T. callipaeda* were included in the study. Cats were randomly allocated to an untreated control group of seven cats or to a Broadline^®^-treated group of eight cats. Cats were treated on Day 0; ocular examinations were performed at inclusion and on Days 7 and 14; eyeworms were recovered and counted on Day 14. The primary efficacy assessment was based on group comparison of number of *T. callipaeda* on Day 14.

**Results:**

Seven days after treatment, six of eight treated cats were negative for eyeworm infection per visual examination, and on Day 14 no eyeworms were found in the treated cats while the seven untreated cats were still infected (geometric mean: 1.97). All cats had inflammatory ocular signs at inclusion; on Day 14, five of eight treated cats had recovered while all untreated control cats were still symptomatic. All collected parasites were confirmed to be *T. callipaeda* by morphology and molecular characterization.

**Conclusions:**

A single treatment with Broadline^®^ provided 100% efficacy against feline thelaziosis and improved related ocular inflammation signs.

**Graphical abstract:**

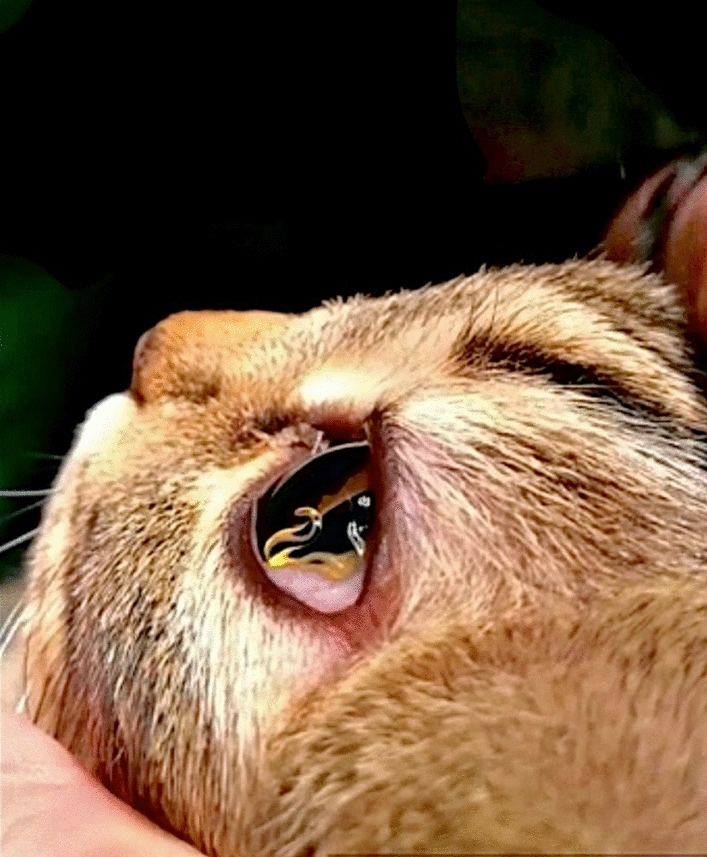

## Background

*Thelazia callipaeda* (Spirurida, Thelaziidae) causes an ocular infection in cats, dogs and other mammals, including humans [1–4]. In definitive hosts, adult parasites (“eyeworms”) reside in the conjunctival sac and underneath the third eyelid [5]. After mating, first-stage larvae (L1) are released in conjunctival secretions by female worms. In Europe, L1 are ingested by the zoophilic males of the fruit fly *Phortica variegata* (Drosophilidae, Steganinae) when feeding on conjunctival fluids. In the intermediate host, L1 develops into infective third-stage larvae (L3) [6]. The transmission to the vertebrate host occurs when the male flies feed again on lachrymal secretions of animals and/or humans [7, 8]. The infection can be subclinical or clinical, and the signs may range from moderate to severe ocular disorders such as epiphora, blepharitis, conjunctivitis, keratitis and corneal ulceration [9]. In recent years, the enzootic areas of *T. callipaeda* infection have expanded from Italy to France, Switzerland, Germany, Spain, Portugal, Bosnia and Herzegovina, Croatia, Romania, Bulgaria, Hungary, Greece, Slovakia and Serbia [4, 8, 10]. Recent increases of clinical cases in pets in Europe have stimulated new studies on the control of the infection. Appropriate treatment and prophylaxis are indeed essential to limit the spread of *T. callipaeda* across Europe and to reduce the risk of zoonotic transmission [4, 11]. Although previous studies have demonstrated the efficacy and safety of topical [12] and oral [13] formulations, therapeutic options for the treatment of feline thelaziosis are limited. The efficacy of the macrocyclic lactone eprinomectin against bovine thelaziosis caused by *Thelazia rhodesi* has been recently demonstrated [14]. The present study aimed at assessing for the first time the efficacy of Broadline^®^, a broad-spectrum parasiticide which comprises eprinomectin, combined with fipronil, (*S*)-methoprene and praziquantel, for the treatment of natural acquired cat thelaziosis.

## Methods

The present trial was a negative controlled, clinical efficacy study with blinding. Group inclusion was carried out using a randomized block design based on order of inclusion. The study protocol was approved by the Italian Ministry of Health with authorization no. 0024844-08/10/2018-DGSAF-MDS-P. The study was performed in two sites in northern Italy (Piedmont region), in an area known to be enzootic for *T. callipaeda* infection in pets [10].

### Inclusion and treatment

Fifteen client-owned cats were included in the study. Cats were of both sexes, aged from 1 to 10 years and weighing 1.7–6.3 kg (Table 1). All cats lived outdoors or were allowed to free-roam.

**Table 1:** Animal and treatment details at inclusion

Group^a^	Case ID	Sex	Body weight (kg)	Applicator size (ml)
1	ITA-01-01	Male	3.1	–
	ITA-01-03	Female	2.9	–
	ITA-01-06	Male	6.3	–
	ITA-01-08	Male	3.0	–
	ITA-01-09	Female	3.2	–
	ITA-02-01	Female	4.0	–
	ITA-02-03	Male	5.1	–
2	ITA-01-02	Female	4.0	0.9
	ITA-01-04	Male	1.7	0.9
	ITA-01-05	Female	3.2	0.9
	ITA-01-07	Female	5.5	0.9
	ITA-01-10	Male	3.7	0.9
	ITA-02-02	Male	4.5	0.9
	ITA-02-04	Male	4.2	0.9
	ITA-02-05	Female	4.0	0.9

^a^Group 1: cats left untreated; Group 2: cats treated with Broadline^®^

On Day 0, cats were clinically examined and weighed. Bilateral ocular examination was carried out to assess the presence of eyeworms and of clinical signs potentially related to thelaziosis (e.g. eye scratching, epiphora, purulent exudation, conjunctivitis, keratitis, blepharospasm and corneal ulceration). When necessary, one drop of local anesthetic (oxybuprocaine hydrochloride 0.4%) was used on the conjunctiva as a common procedure in ophthalmologic examinations. The infection level was estimated (1 worm, 2–5 worms, > 5 worms) by visual inspection of the conjunctival sac in both eyes (Table 2).

**Table 2:** Individual level of *Thelazia callipaeda* infection on Day 0 and Day 7 and count after flushing on Day 14

Group^a^	Case ID	Day 0		Day 7		Day 14		
		Left	Right	Left	Right	Left	Right	Total
1	ITA-01-01	2–5	1	2–5	1	2	1	3
	ITA-01-03	2–5	2–5	2–5	2–5	2	4	6
	ITA-01-06	1	1	1	1	1	1	2
	ITA-01-08	0	1	0	1	0	1	1
	ITA-01-09	1	0	1	0	1	0	1
	ITA-02-01	2–5	1	2–5	2–5	1	1	2
	ITA-02-03	1	2–5	1	1	0	1	1
2	ITA-01-02	0	2–5	0	0	0	0	0
	ITA-01-04	1	2–5	0	0	0	0	0
	ITA-01-05	2–5	2–5	0	0	0	0	0
	ITA-01-07	1	0	1	0	0	0	0
	ITA-01-10	2–5	1	0	0	0	0	0
	ITA-02-02	1	1	0	0	0	0	0
	ITA-02-04	2–5	2–5	0	0	0	0	0
	ITA-02-05	2–5	1	1	0	0	0	0

^a^Group 1: cats left untreated; Group 2: cats treated with Broadline^®^

After inclusion, cats were allocated into the untreated control, Group 1 (G1), or into Group 2 (G2), treated once with Broadline^®^ according to label instructions. Seven cats were enrolled in G1 and eight cats in G2.

### Post-treatment evaluations and efficacy criteria

On Days 7 and 14 (± 1), cats were assessed for the presence of *T. callipaeda* by ocular examination and for clinical signs associated with thelaziosis.

On Day 14, the conjunctival fornix was flushed with ~ 5 ml of saline solution (0.9%) for parasite recovery. Eyeworms were removed, counted separately for each eye and stored in 70% ethanol. All nematodes were identified based on key morphological features [15, 16]. Identification was also confirmed by a PCR specific for a 689-bp-long portion of the gene encoding for the mitochondrial cytochrome *c* oxidase subunit 1 (*cox*1) using the primer set NC1–NC2 [17]. PCRs were carried out as previously described [18]. Amplicons were purified and sequenced, and then sequences were determined in both directions and the electropherograms verified by eye with Chromas Lite software. The sequences were aligned using the Data Analysis in Molecular Biology program and Evolution version 4.5.55 (DAMBE) and compared with those available in the GenBank™ using the Basic Local Alignment Search Tool (BLAST).

The primary efficacy criterion for the assessment of curative efficacy was the number of *T. callipaeda* worms in G2 compared to G1 on Day 14. Counts were transformed to the natural logarithm (ln) of (count + 1) for calculation of geometric means for each treatment group (geometric mean = exp (average of ln (worm count + 1) within treatment group) – 1). Percent efficacy was calculated using the following formula: 100 × [(*C* − *T*)/*C*], where *C* is the geometric mean among G1 and *T* is the geometric mean among G2. The un-transformed counts in G2 were compared to the counts in G1 using a non-parametric Wilcoxon rank sum test.

In addition, the proportion of cats positive for *T. callipaeda* worms in the Broadline^®^-treated group compared to the control group was analyzed. A cat was considered positive if at least one worm was counted in at least one eye on Day 14. The comparison was performed using Fisher’s exact test. The worm-free efficacy (%) was calculated at each time point using the formula: efficacy (%) = ((proportion of positive animals in the control group – proportion of positive animals in the IVP-treated group)/proportion of positive animals in the control group)) × 100. Proportions were used as the number of cats was different in both groups.

All testing was two-sided at significance level *α* = 0.05.

All cats completed the study according to the protocol and were included in the efficacy calculations. After the end of the study, the control cats harboring eyeworms received an appropriate curative treatment.

## Results

At the end of the study (i.e. on Days 14 ± 1), all cats in the control group were still infected with *T. callipaeda* (geometric mean = 1.97) while all cats in G2 were free of infection. There were significantly fewer worms in G2 compared to G1 (*p* < 0.05) (Tables 2, 3) resulting in a 100% efficacy rate.

**Table 3:** Efficacy based on worm count (geometric mean) on Day 14

Group^a^	Worm count
1 (*n* = 7)	1.97
2 (*n* = 8)	0.0
*p*-value^1^	0.00052
Efficacy (%)	100

^a^Group 1: cats left untreated; Group 2: cats treated with Broadline^®^

^1^*p*-value: Wilcoxon rank sum test

On Day 7, six of eight cats in the treated group were negative for eyeworm infection while all cats were negative on Day 14 (Table 2). Efficacy based on the proportion of infected cats was 75% and 100% on Days 7 and 14, respectively, with significantly fewer infected cats in G2 compared to G1 (*p*-value < 0.05) (Table 4).

**Table 4:** Efficacy based on the number of infected cats on Days 7 and 14

Group^a^	Day 7	Day 14 (± 1)
1 (*n* = 7)	7	7
2 (*n* = 8)	2	0
*p*-value^1^	0.000013	0.000105
Efficacy (%)	75	100

^a^Group 1: cats left untreated; Group 2: cats treated with Broadline^®^

^1^*p*-value: Fisher’s exact test

The identity of the worms was confirmed morphologically and molecularly. All samples produced PCR products of the expected size. Generated sequences showed 100% homology with *T. callipaeda* GenBank accession number AM042549.1 (haplotype 1).

At enrollment, all cats showed ocular clinical signs compatible with thelaziosis. At study end, all cats in G1 still showed clinical signs (Table 5). In G2, five cats recovered clinically after the treatment while three remained symptomatic (Table 5). Specifically, one cat that showed epiphora/lacrimation, purulent exudation, conjunctivitis, keratitis and blepharospasm at inclusion presented eye scratching, epiphora/lacrimation, purulent exudation, conjunctivitis and keratitis on Day 7 and eye scratching, epiphora/lacrimation and conjunctivitis on Day 14. The second cat showed eye scratching, epiphora/lacrimation, conjunctivitis and blepharospasm at inclusion and epiphora/lacrimation and conjunctivitis on Days 7 and 14. The third cat showed epiphora/lacrimation, purulent exudation, conjunctivitis and blepharospasm at day 0, eye scratching and conjunctivitis at day 7 and only conjunctivitis at day 14.

**Table 5:** Number (*n*/tot) and percentage (%) of cats infected with *Thelazia callipaeda* showing one or more ocular clinical signs on Days 0, 7 and 14 (± 1)

	G1			G2		
	Day 0	Day 7	Day 14 (± 1)	Day 0	Day 7	Day 14 (± 1)
Eye scratching	3/7 (42.9)	3/7 (42.9)	2/7 (28.6)	2/8 (25)	3/8 (37.5)	1/8 (12.5)
Epiphora/lacrimation	6/7 (85.7)	6/7 (85.7)	6/7 (85.7)	8/8 (100)	5/8 (62.5)	2/8 (25)
Purulent exudation	3/7 (42.9)	3/7 (42.9)	3/7 (42.9)	4/8 (50)	1/8 (12.5)	0/8 (0)
Conjunctivitis	5/7 (71.4)	6/7 (85.7)	6/7 (85.7)	8/8 (100)	7/8 (87.5)	3/8 (37.5)
Keratitis	0 (0)	0 (0)	0 (0)	2/8 (25)	2/8 (25)	0 (0)
Blepharospasm	2/7 (28.6)	2/7 (28.6)	2/7 (28.6)	5/8 (62.5)	1/8 (12.5)	0/8 (0)
Total number of cats with clinical signs	7/7 (100)	7/7 (100)	7/7 (100)	8/8 (100)	8/8 (100)	3/8 (37.5)

Group 1: cats left untreated; Group 2: cats treated with Broadline^®^

Four cats (2 in G1 and 2 in G2) received concomitant treatments during the study (i.e. antibiotics and/or anti-inflammatory drugs) to manage conjunctivitis related to eyeworm infection.

No adverse events occurred during the study.

## Discussion

The present study is the first clinical trial evaluating the efficacy of the topical combination containing fipronil/(*S*)-methoprene/eprinomectin and praziquantel against *T. callipaeda* in naturally infected cats. A single administration of the product was fully efficacious and safe for the elimination of eyeworms within 14 days.

Feline thelaziosis poses important challenges in feline medicine practice because cats may be infected subclinically or show only mild clinical signs overlapping those of other common feline ocular diseases such as ocular viral and/or bacterial infections, which can also secondarily occur in infected cats because of discomfort-caused itching and scratching [9, 19]. Moreover, clinical signs and the presence of eyeworms can be overlooked by veterinarians because in-depth ophthalmic examination in cats commonly requires sedation or general anesthesia as performed for some of the study cats. When thelaziosis is diagnosed, a specific treatment is needed. Therapeutic options for feline ocular thelaziosis in Europe are limited to mechanical removal of the parasites from the eyes or to the administration of some antiparasitic drugs containing macrocyclic lactones. The therapeutic efficacy of an oral dewormer containing milbemycin oxime and a topical containing moxidectin has been demonstrated [12, 13]. Oral milbemycin oxime proved 53.3% and 73.3% effectiveness after one or two treatments at weekly intervals, respectively [13]. The efficacy of the single administration of the spot-on formulation containing moxidectin and imidacloprid was 93.3% and 100% 14 and 28 days post-treatment, respectively [12]. The latter is approved in the EU for treating *T. callipaeda* infection in cats.

Now, the administration of Broadline^®^ has also demonstrated 100% efficacy in the treatment of cats who were cleared of *T. callipaeda* within 2 weeks. The post-treatment clinical recovery of five cats in 14 days and the significant reduction of the clinical signs in three treated animals further support the usefulness of this formulation in feline clinical practice. According to the data generated herein, the concomitant administration of anti-inflammatory drugs could be of benefit to obtain a faster clinical recovery in severely infected cats and to guarantee animal welfare.

In general, feline vector-borne diseases are often underestimated, and there is a need to increase awareness on their importance for cats [20–22]. Accordingly, correct diagnosis and effective treatments of cats infected with vector-borne pathogens, such as *T. callipaeda*, should be implemented to limit their spreading and the risk of infections for both animals and humans. Although cases of infection by *T. callipaeda* in humans have been reported from different European countries (e.g. Italy, France, Spain, Serbia, Croatia and Germany), human thelaziosis in this continent is still regarded as a rare disease [4]. Considering the zoonotic nature of the infection, preventive measures should also be implemented in enzootic areas to reduce the occurrence of the disease. The monthly administration of antiparasitic drugs containing macrocyclic lactones, i.e. oral milbemycin oxime (0.5 mg/kg b.w.) (NexGard^®^ Spectra) and moxidectin spot-on (2.5%) (Advocate^®^), is effective in preventing thelaziosis in dogs [23, 24]. Considering that the topical combination containing fipronil, (*S*)-methoprene, eprinomectin and praziquantel is highly efficacious against developing stages of lungworms and heartworms [25–27], its efficacy in preventing the establishment of adult *Thelazia* in cats is worth of further studies.

## Conclusions

In the present study, a single treatment with Broadline^®^ provided 100% efficacy against *Thelazia* infection in cats and improved thelaziosis-related ocular inflammation signs.

## Data Availability

All relevant data are included within the article.

## Abbreviations

L1: First-stage larvae
L3: Third-stage larvae
G1: Group 1, cats left untreated
G2: Group 2, cats treated with Broadline^®^
PCR: Polymerase chain reaction
